# Treatment of Puerperal Eclampsia

**Published:** 1877-05-20

**Authors:** 


### TREATMENT OF PUERPERAL ECLAMPSIA.

Smith Baker, M. D., of Whitesboro, N. Y., in Chicago Medical Journal and Examiner, says:

"At a preliminary examination, to be held from two to four weeks before the expected labor, determine so far as possible whether there be (*a*) hyperæmia; if so, prescribe a low diet, saline cathartics, and potassic bromide thrice daily; (*b*) if there be anæmia, order a good diet, tr. ferri chlor. after meals, and an aperient pill at bed-time, if necessary; (*c*) if œdema or albuminuria exist, these will need the exhibition of tr. ferri chlor. with meals, and potassic iodide between meals and at bed-time; (*d*) headache, malaise, irritability, etc.; for these remove the cause, if possible, and if necessary, give sufficient opiate to effect relief.

"During labor, if convulsions occur, seek out the special conditions of the patient, and act as indicated. A full, strong, bounding pulse, and a bright-colored mucous membrane will require venesection carried to the extent of securing its sedative effect, a half-drachm dose of chloral per mouth or rectum, means for inducing a movement of the bowels, and a free perspiration, relief of the bladder if distended, and the exhibition of chloroform for anticipating and controlling the convulsions. A feebler pulse, pale membranes, and œdema will, as a rule, require or permit an active cathartic only, and chloral and chloroform. If the convulsions recur—unless it be previous to the seventh month, when repeated hypodermic injections of morphine should be given, and the labor left to nature for a longer time—proceed quickly but gently to dilate the os uteri by means of tents and Barnes' dilators, puncture the membranes, excite uterine contractions by means of warm uterine douche and ergot, and deliver as soon as possible.

"After the delivery is accomplished let the uterine hemorrhage proceed moderately, inject hypodermically one sixth to one quarter grain of morphia, and give broths and stimulants as required; exercise extreme care about the after attentions, as bathing, etc., and prohibit and prevent all disturbances by friends or otherwise.

"In fine, by food, tonics, and stimulants, by purging, bleeding, or sweating, by quietness or delivering, by opium, bromides, chloral, and chloroform, each in its time to suit the peculiar circumstances of individual cases, and guided by judgment unobscured by theories, we may be more successful than hitherto at the bedside of our patients with puerperal eclampsia."—*Louisville Medical News.*

